# Machine learning applications for early detection of esophageal cancer: a systematic review

**DOI:** 10.1186/s12911-023-02235-y

**Published:** 2023-07-17

**Authors:** Farhang Hosseini, Farkhondeh Asadi, Hassan Emami, Rayan Ebnali Harari

**Affiliations:** 1 Department of Health Information Technology and Management, School of Allied Medical Sciences, Shahid Beheshti University of Medical Sciences, Tehran, Iran; 2 Department of Emergency Medicine, Harvard Medical School, Boston, MA USA

**Keywords:** Machine learning, Deep learning, Esophagus, Esophageal Cancer, Early detection

## Abstract

**Introduction:**

Esophageal cancer (EC) is a significant global health problem, with an estimated 7th highest incidence and 6th highest mortality rate. Timely diagnosis and treatment are critical for improving patients’ outcomes, as over 40% of patients with EC are diagnosed after metastasis. Recent advances in machine learning (ML) techniques, particularly in computer vision, have demonstrated promising applications in medical image processing, assisting clinicians in making more accurate and faster diagnostic decisions. Given the significance of early detection of EC, this systematic review aims to summarize and discuss the current state of research on ML-based methods for the early detection of EC.

**Methods:**

We conducted a comprehensive systematic search of five databases (PubMed, Scopus, Web of Science, Wiley, and IEEE) using search terms such as “ML”, “Deep Learning (DL (“, “Neural Networks (NN)”, “Esophagus”, “EC” and “Early Detection”. After applying inclusion and exclusion criteria, 31 articles were retained for full review.

**Results:**

The results of this review highlight the potential of ML-based methods in the early detection of EC. The average accuracy of the reviewed methods in the analysis of endoscopic and computed tomography (CT (images of the esophagus was over 89%, indicating a high impact on early detection of EC. Additionally, the highest percentage of clinical images used in the early detection of EC with the use of ML was related to white light imaging (WLI) images. Among all ML techniques, methods based on convolutional neural networks (CNN) achieved higher accuracy and sensitivity in the early detection of EC compared to other methods.

**Conclusion:**

Our findings suggest that ML methods may improve accuracy in the early detection of EC, potentially supporting radiologists, endoscopists, and pathologists in diagnosis and treatment planning. However, the current literature is limited, and more studies are needed to investigate the clinical applications of these methods in early detection of EC. Furthermore, many studies suffer from class imbalance and biases, highlighting the need for validation of detection algorithms across organizations in longitudinal studies.

## Background

Esophageal cancer (EC) is a malignant neoplasm arising from the esophagus tissues and is classified into two most common forms: esophageal adenocarcinoma (EAC) and esophageal squamous cell carcinoma (ESCC) according to the National Cancer Institute’s definition [1]. As the 7th most common cancer in the world and the 6th leading cause of cancer-related death, its incidence is expected to increase by 140% in the next few years [2]. The burden of EC is considerably higher in less developed regions, where approximately 80% of cases occur. Roughly 70% of cases are found in males, and there is a 2 to 5-fold higher incidence and mortality rate between the genders, which increases with age [3]. Esophageal malignancies have a grim prognosis due to their tendency to remain asymptomatic, leading to late-stage diagnosis. Consequently, definitive resection and treatment are often not viable options. More than half of the cases involve distant metastases or unresectable disease, resulting in a discouraging 5-year survival rate of only 18%, albeit showing slight improvement over time. Considering the weak correlation between esophageal symptoms and cancer or precursor lesions, screening and monitoring for EC pose significant challenges. In fact, most patients diagnosed with early-stage EC exhibit no symptoms until the onset of dysphagia and weight loss, which could indicate an advanced tumor. However, in cases where EC is detected early, evolving therapies not only enhance cure rates but also reduce treatment-related complications [4].

The incidence of EC has exhibited a significant surge on a global scale in recent times. Based on the GLOBOCAN 2020 report, if the present trends continue, the anticipated figures of EC occurrences and fatalities in 2030 and 2040 can be estimated by multiplying the 2020 rate with the anticipated populace in 2030 and 2040. It is predicted that by 2030, the number of fresh cases of EC will reach 739,666, and the associated deaths will amount to 723,466 [3].

Endoscopy is a primary diagnostic tool to determine the presence and location of EC, the distance between the cancer and the tooth, the length of the tumor [5], the degree of peripheral involvement [6], the degree of obstruction [7], and the presence of mucous nodes [8]. However, these symptoms are not always easy to detect [9], and an accurate diagnosis requires experienced physicians. Several studies have shown that it is often possible to miss symptoms and suspicious areas during endoscopy [10]. Therefore, suspected patients should be regularly followed up through endoscopic examination to control the progress of abnormalities in the next stages [11, 12].

### Machine learning in EC

As the number of patients with EC continues to grow, computer-aided diagnosis (CAD) systems have attracted increasing attention [13]. Recent advancements in AI have shown promising applications in diagnostic imaging in various medical fields [14–16]. AI is a general term that refers to a wide range of algorithms capable of identifying features among a large amount of data to provide clinical inference and insights. Machine learning (ML) is a subset of AI and refers to algorithms that can learn and predict with or without explicit instructions [17]. ML in medical imaging can improve decision making and diagnosis time by providing reliable clinical decision support. The most important characteristic of a ML model is to adapt independently, learn from previous calculations and produce reliable results when new datasets are exposed to models repeatedly [18–20].

With the surge of medical imaging for screening esophagus tissues, a large volume of imaging data with various characteristics including type and stage of EC and patients’ complications is produced every day. This amount of data can be a great resource for better understanding underlying factors of EC, early detection, and ultimately timely diagnosis of EC. On the other hand, monitoring and analyzing this type of massive imaging data is beyond the capability of humans. To fill this gap, several previous studies have applied ML-based methods in early detection and diagnosis of EC. However, our understanding regarding the performance of these ML-based modeling approaches in EC is still very limited. The aim of this study was to systematically review the scientific literature and describe how ML algorithms have been applied to the early detection of EC. Additionally, we aimed to discuss the methodological and design characteristics of the existing studies in this realm, informing future research and development efforts on using ML methods to improve patients’ outcomes and reduce the burden of costs for patients, organizations, and insurances. Specifically, this study aims to answer the following research questions:

1. To what extent has ML been effective in the early detection of EC?
2. Which ML algorithms have demonstrated superior performance in the analysis of esophagus-related images?

This systematic review has been conducted to address research questions related to the development of more accurate and efficient diagnostic tools for a particular medical condition. The review methodology involved a rigorous search of the literature to identify relevant studies, followed by a systematic and thorough extraction and analysis of the data. The results section of the review provides a detailed examination of the target population, dataset quantity and characteristics, and algorithms employed in the reviewed articles. The algorithms are categorized based on the methodologies used in the respective articles, and specific details are analyzed and explained. In the discussion section of the review, the obtained results are compared and elucidated, with a focus on highlighting noteworthy aspects, challenges, weaknesses, and strengths of both the articles and the utilized algorithms. This synthesis of the data from the included studies provides a comprehensive and rigorous analysis of the current state of knowledge on the topic, and has the potential to inform the development of more accurate and efficient diagnostic tools, ultimately improving patient outcomes.

## Methodology

### Search strategy

A systematic search strategy was developed based on previous studies and criteria selected by the authors. All articles that used ML methods for the early detection of EC were reviewed. A comprehensive search was conducted in PubMed, Scopus, Web of Science, Wiley, and IEEE databases using keywords such as ML, Deep Learning (DL), Neural Networks (NN), esophagus, EC, and early detection, based on inclusion and exclusion criteria from 2018 to December 10, 2022. Related articles were extracted from these databases.

### Eligibility of studies

**Fig. 1:**
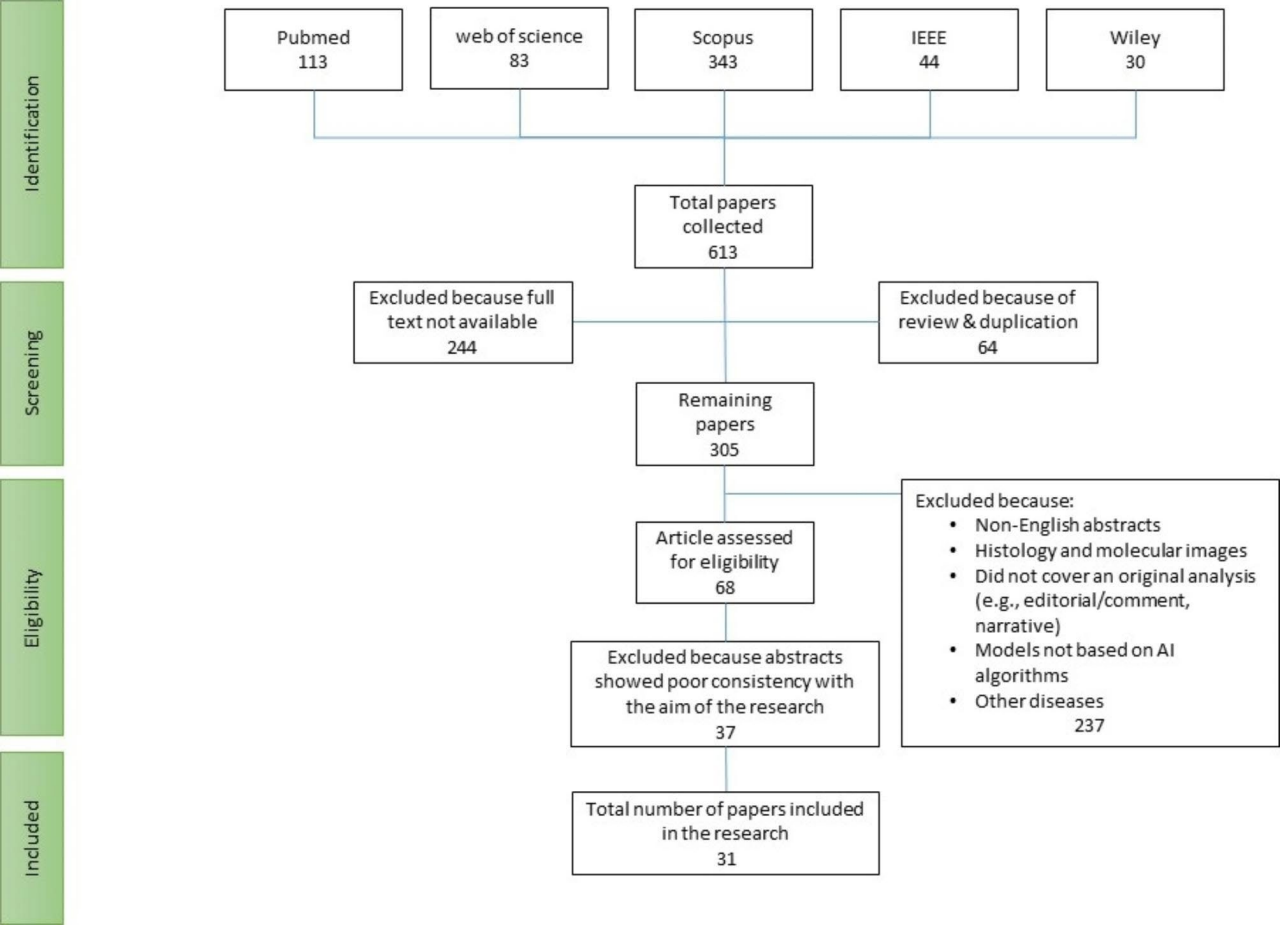
Flow diagram of studies identified in the systematic review

The inclusion and exclusion criteria for the systematic review were carefully defined. The inclusion criteria were as follows: [1] studies that used ML methods for the early detection and classification of EC, [2] studies written in English, [3] full-text articles available, and [4] studies published in the last 5 years. Any study that met all of the above criteria was selected for review. The exclusion criteria included: [1] studies related to other diseases, [2] studies published in a non-English language, [3] studies that used other imaging modalities except for endoscopy, and [4] review, meta-analysis, and narrative studies. Any study that met at least one of the above criteria was excluded from the systematic review. The process of study selection is presented in a PRISMA flowchart, as shown in Fig. 1.

### Data extraction

In this systematic review, the process of data extraction involved a thorough examination of previous articles to gather information regarding their methods and results. The articles were extracted using standardized table formats, encompassing the following elements:

- Article title.
- Country where the study was conducted.
- Year of publication.
- Type of ML methods utilized.
- Studied society.
- Accuracy, sensitivity, and specificity of the ML algorithm.
- Modality.
- Cancer type.

The methodology used in this study involved a rigorous and systematic approach to identifying and selecting relevant articles for inclusion. The process began with a review of the abstracts of all relevant articles, which were then input into Endnote for further analysis. Next, the research team assessed the title, abstract, and keywords of each article, applying inclusion and exclusion criteria to select studies that met the predetermined standards for quality and relevance. Duplicates were removed, and the research team performed full text review of the selected papers. To ensure the accuracy and validity of the data extraction process, a designated data extraction form was used, which had been confirmed for validity by two medical informatics experts. The full text of each article was reviewed by two researchers, and data were collected using this form. Finally, all findings obtained from the data extraction form were reviewed and validated by a third reviewer. Summary of the results is presented in Table 1.

**Table 1:** Characteristics of studies for early detection of EC using ML

Title	Algorithm	Image	Patient	Country
Lou et al. 2020	U-Net	80	-	China
Ghatwary et al. 2019	Faster R-CNN Single-Shot Multibox Detector	100	39	UK
Tang et al. 2022	multi-task classification and segmentation)MTCS(	805	255	China
Ghatwary et al. 2019	Faster R-CNN	1000	-	UK
Yu et al. 2021	Multi-task learning (MTL)	1003	-	Taipa
Wu et al. 2021	Faster-RCNN Dual-Stream Network (DSN)	1051	-	China
Liu et al. 2020	Convolutional Neural Networks (CNN)	1272	748	China
Groof et al. 2020	hybrid ResNet-Unet	1704	669	Netherland
Tang et al.2021	DCNN	4002	1078	China
Meng et al. 2022	YOLO v5	4447	837	China
Gong et al. 2022	“Neuro-T” version 2.3.2	5162	-	Korea
Shiroma et al.2021	Single Shot MultiBox Detecto	8428	-	Japan
Du et al. 2021	random weighted sampling (RWS)	20,965	4,077	China
Putten et al. 2020	U-Net	494,356	-	Netherland
Gan et al. 2020	dual-stage U-shape convolution network (D-UCN)	-	-	China
Wang et al. 2021	Cascade RCNN	-	80	China
Sui et al. 2021	V-Net	-	414	China
Takeuchi et al. 2021	VGG16	-	457	Japan
Ghatwary et al. 2021	3DCNN	-	-	UK
Alharbe et al. 2022	Deep transfer learning	-	-	Saudi Arabia
Zhao et al. 2022	Google Net V3 TensorFlow 1.6	-	300	China
Collins et al. 2021	SVM, MLP, 3DCNN	-	10	France
Zhao et al.2021	CNN	-	500	China
Chen et al.2021	Faster RCNN	1520	421	China
Tsai et al.2021	Single Shot MultiBox Detector	155	-	Taiwan
Tsai et al.2022	single-shot multi-box detector	1780	-	China
Sali et al.2020	ResNet34	387	130	USA
Wang et al. 2021	single-shot multibox detector	498	-	Taiwan
Zhang et al. 2022	Faster R-CNN VGG16	6445	200	China
Guo et al.2020	SegNet	6473	-	China
Fang et al. 2022	U-Net	75	-	Taiwan

## Results

**Fig. 2:**
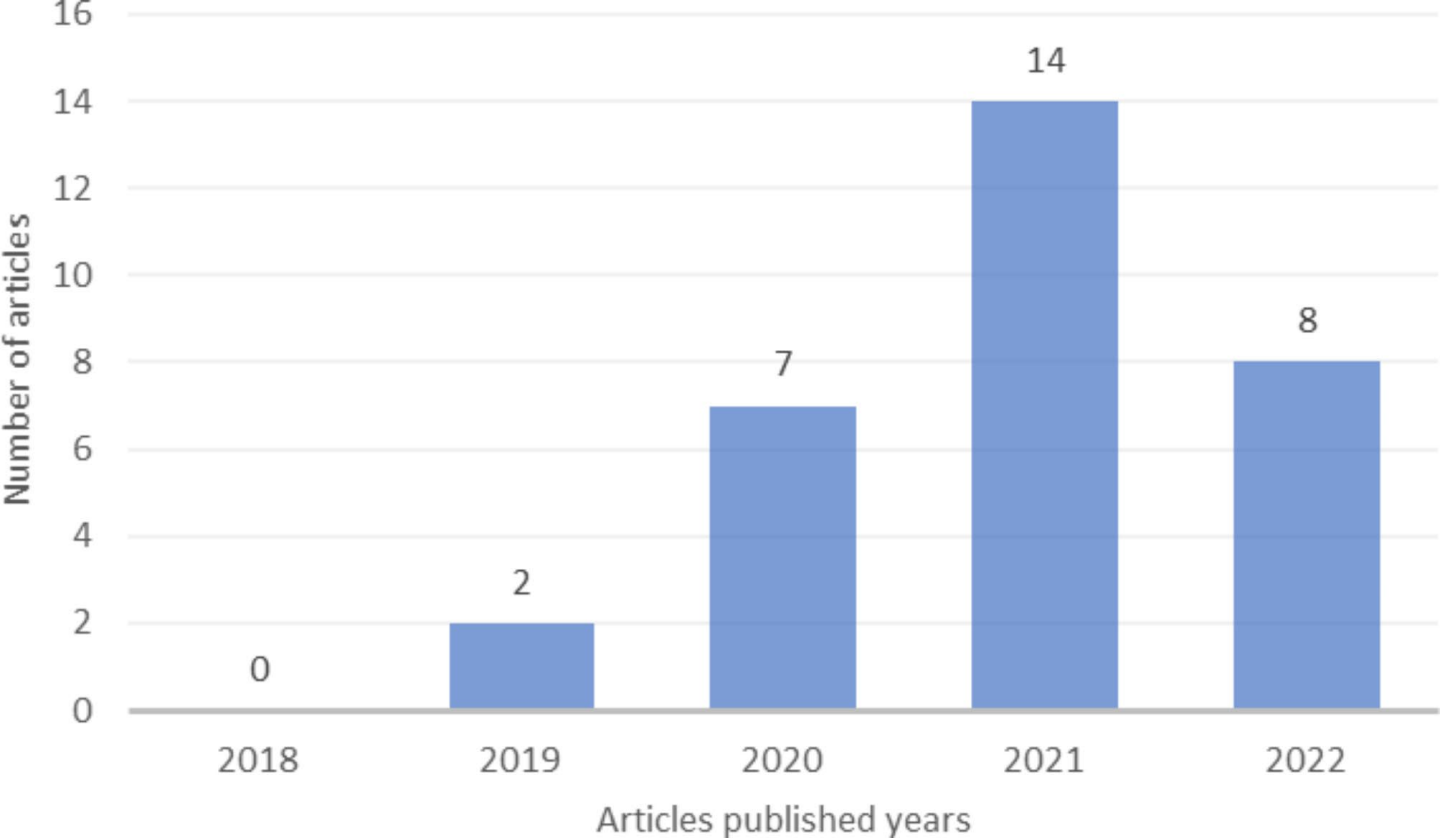
Number of Papers Published from January 2018 to December 2022

**Fig. 3:**
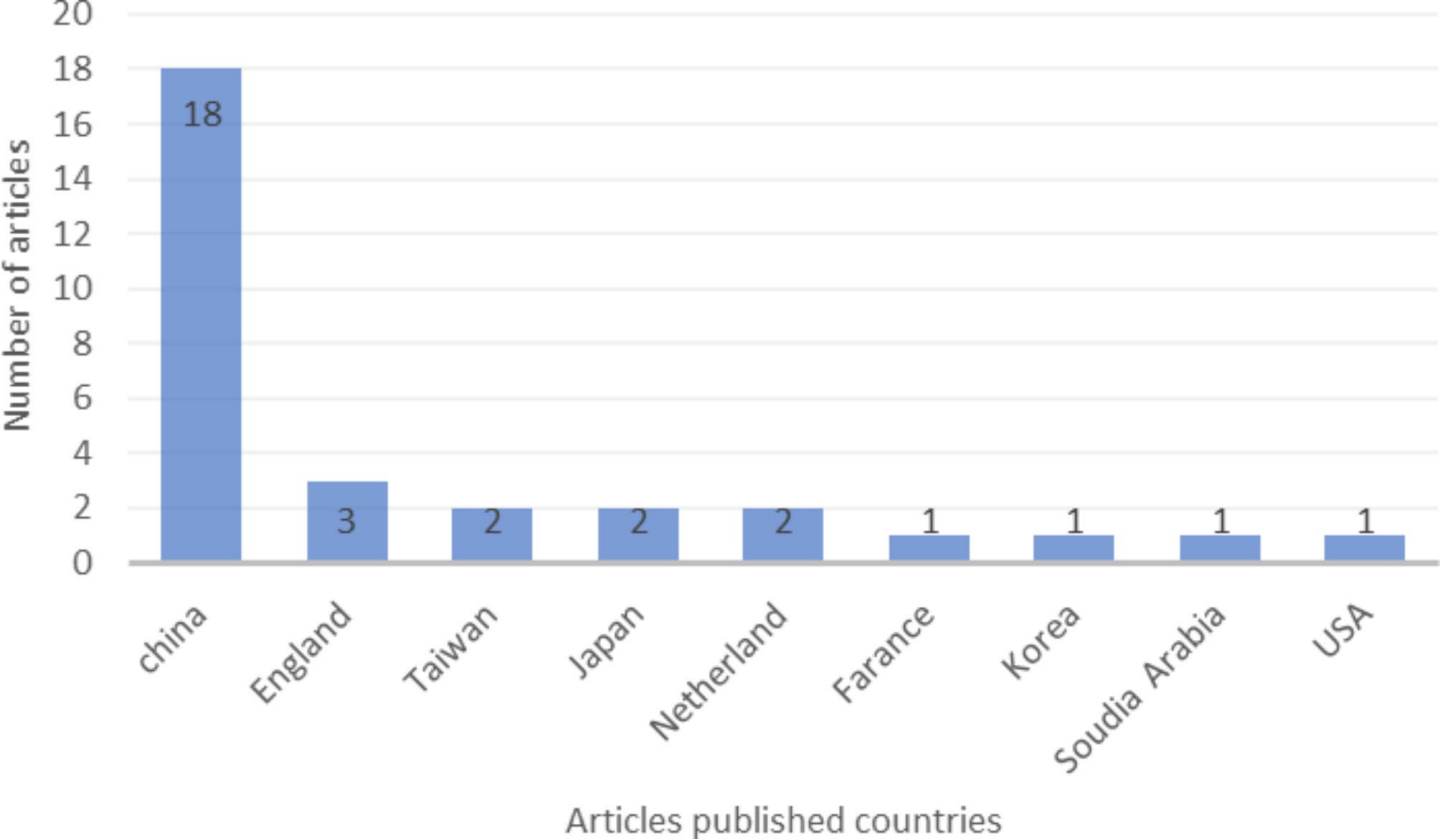
Number of articles published based on country

After an initial search of five databases, a total of 613 articles were identified. By screening the titles and abstracts, 56 articles were selected for full-text review. After applying inclusion and exclusion criteria, 31 articles were ultimately included in the systematic review. Based on the inclusion criteria, a total of 31 articles were selected and reviewed. The analysis showed that the majority of articles were published in the past two years, with 14 articles in 2021 and 8 articles in 2022. Furthermore, 7 articles were published in 2020 and 2 articles in 2019, while no eligible articles were identified in 2018 (Fig. 2). In addition, the frequency of articles by country of origin was also examined, with the highest number of articles being published by research teams from China, followed by England (Fig. 3).

The review synthesizes the findings from previous studies in the field and is organized into six sections as follows:

### Characteristics of EC image datasets

**Fig. 4:**
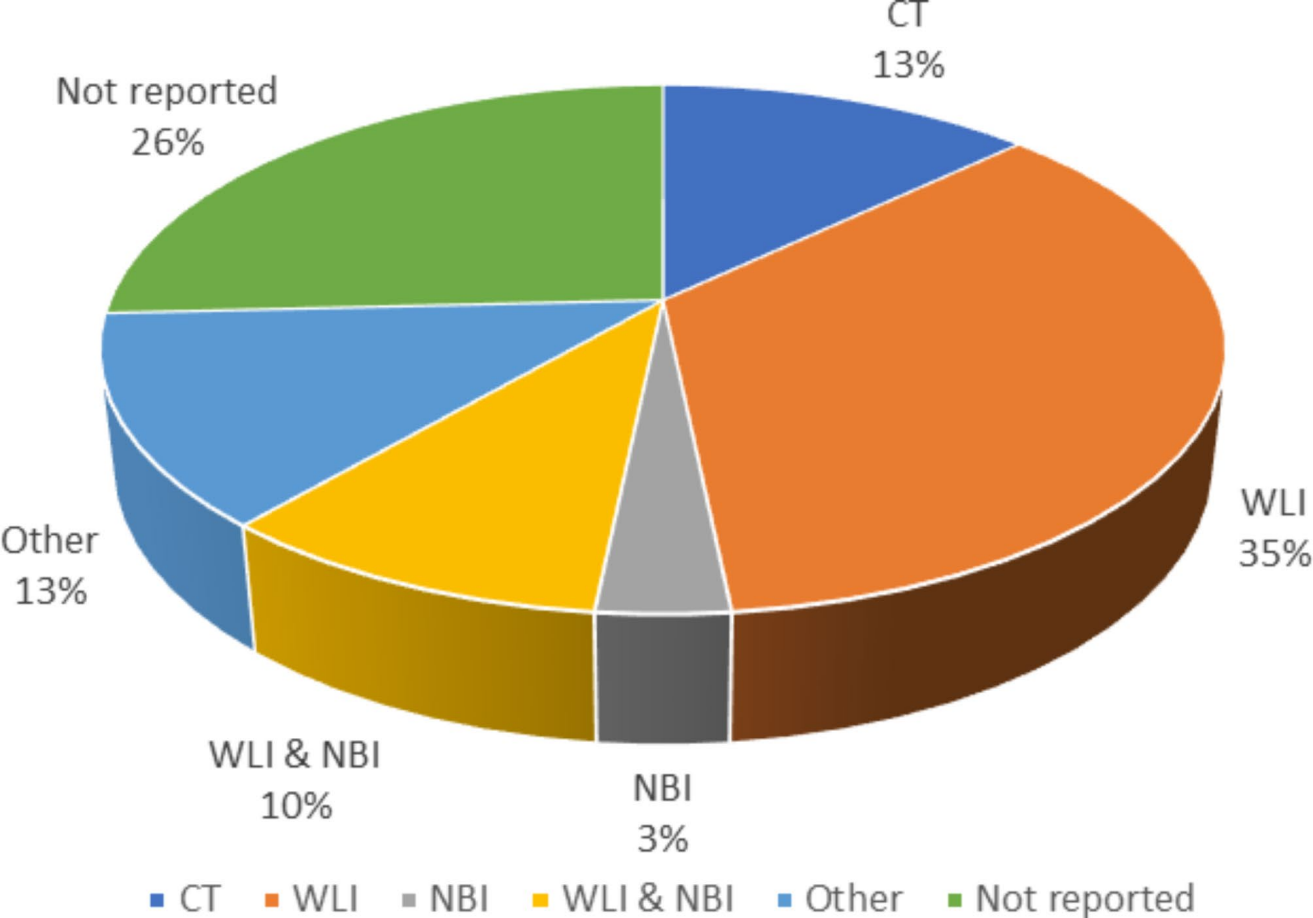
Frequency of modalities used in ML methods to detection of EC

**Fig. 5:**
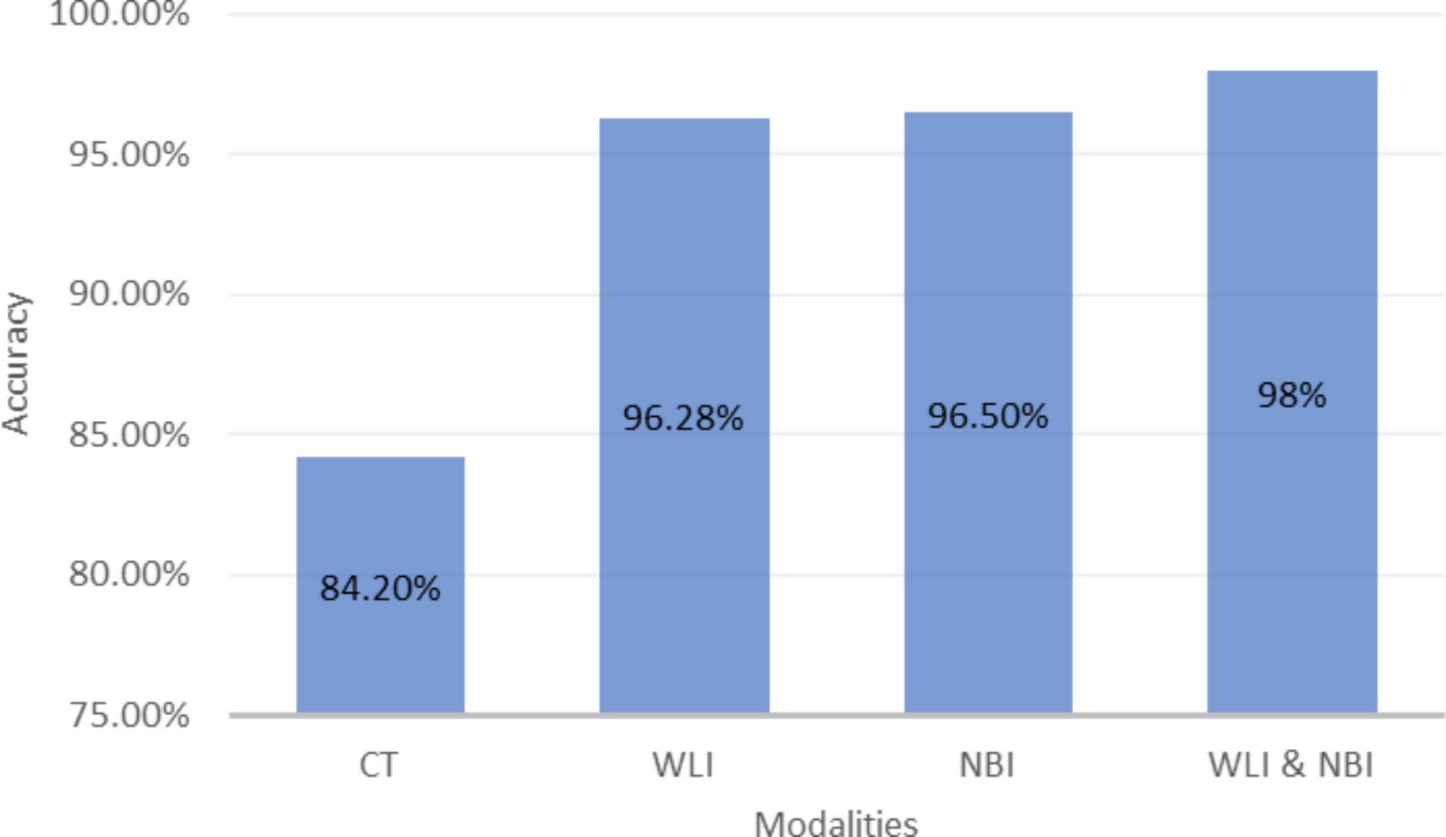
Accuracy of modalities used in ML methods to Detection of EC

In early detection of EC, different imaging modalities such as gastroscopy, white light imaging (WLI), and narrow-band imaging (NBI) have been used in various studies [21–23]. A review of the literature showed that WLI images were used in 35% of studies [13, 19, 24–35], followed by a combination of WLI and NBI images in 10% [20, 25, 36, 37], computed tomography (CT) images in 13% [38–41], NBI images in 3% [42], images of other modalities in 13% [43–46], and the type of imaging was not mentioned in 26% of studies (Fig. 4) [34, 44, 47–52]. It was also observed in the survey that the highest average accuracy (98%) among the types of modalities used is related to the algorithms that used a combination of WLI and NBI [20, 25, 36, 37]. Among the algorithms that used only one type of modality, the average accuracy was 96.5%, which was related to NBI [42], and then 96.3% and 84.2% were related to WLI [13, 26–30, 32, 33] and CT [40, 41], respectively. In one case, an average accuracy of 98% was achieved using Optical coherence tomography (OCT) images (Fig. 5) [39].

Most studies used locally collected datasets, and only three studies used known datasets in the field of clinical images such as MICCAI version 2015, Kvasir Dataset, and ImageNet [13, 36, 48]. Various ML techniques were employed for data recognition and classification, with the maximum number of images used for early detection of EC through ML algorithms being 494,356 images [35], and the least used image being 80 images [36, 38]. On average, 28,939 images were used in the field of EC detection.

### Characteristics of ML algorithms

**Fig. 6:**
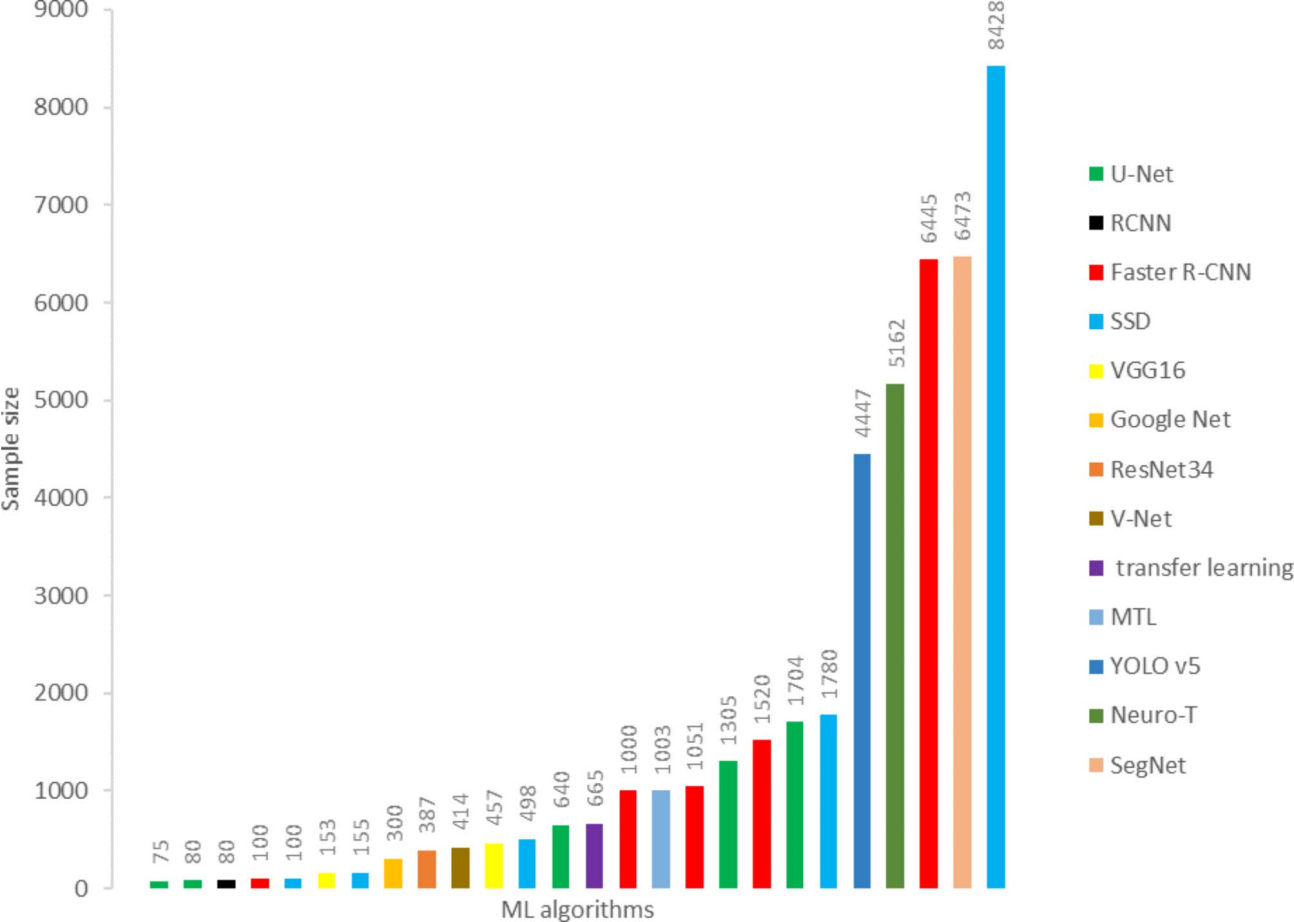
The sample size used in ML algorithms

**Fig. 7:**
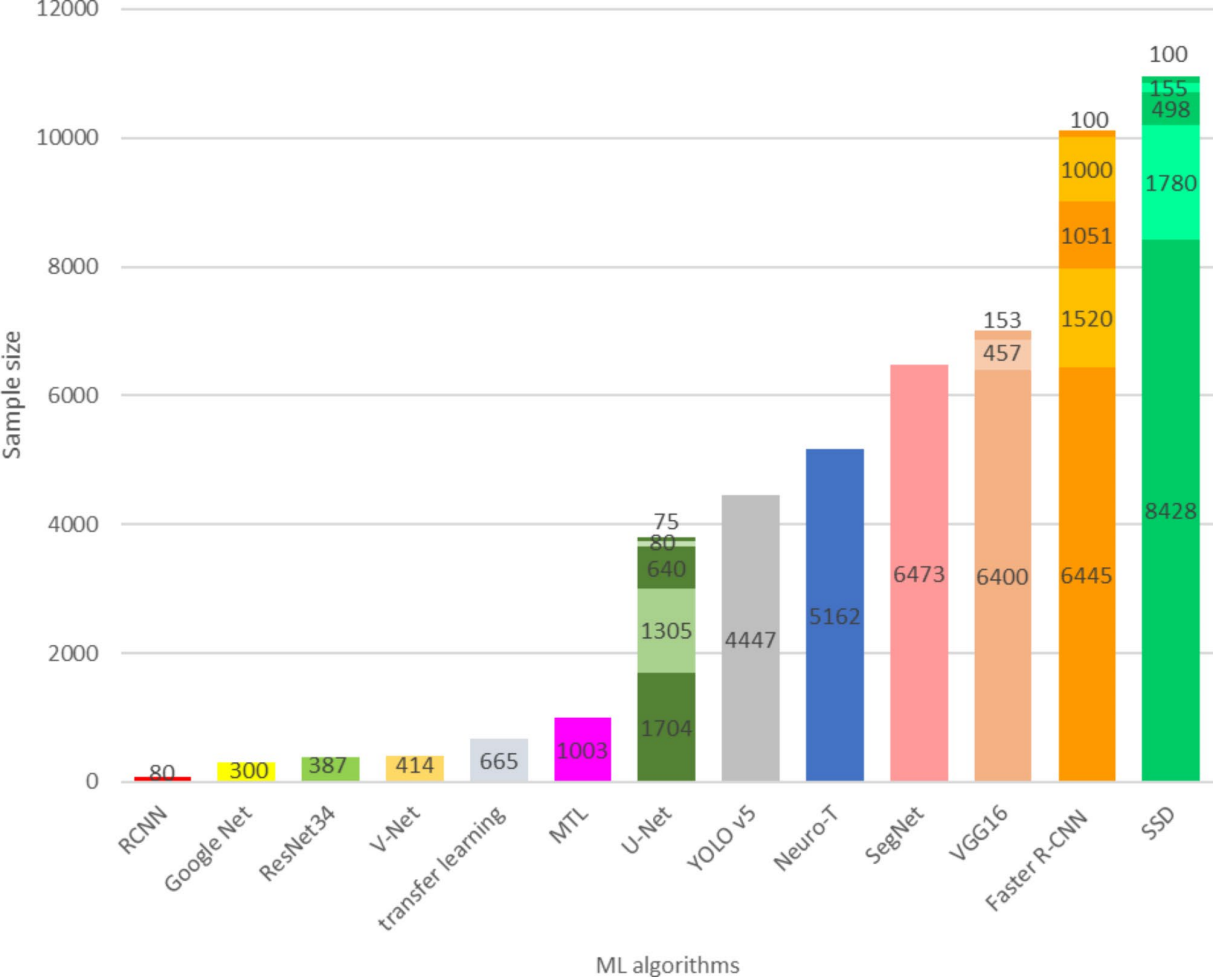
The sample size used in ML algorithms

Our review of the literature showed that among all of the algorithms used in the studies, the Single-Shot Multibox Detector (SSD) algorithm had the largest sample size [20]. Furthermore, Faster R-CNN (6445 images), SegNet (6473 images), Neuro_T (5162 images), and YOLO v5 (4447 images) were other ML algorithms that utilized a large sample size for training, testing, and validation [24, 27, 42, 49]. In addition, in studies focused on early detection of esophageal cancer, U-Net [33, 35, 36, 38, 39], Faster R-CNN [13, 26, 48, 49, 51] SSD [13, 20, 25, 30, 37] algorithms reported in 5 studies had the highest number of uses among all ML algorithms. VGG16 algorithm was also used in 3 studies [25, 28, 49]. Details of the algorithms and the sample sizes used in the studies can be found in Figs. 6 and 7.

**Fig. 8:**
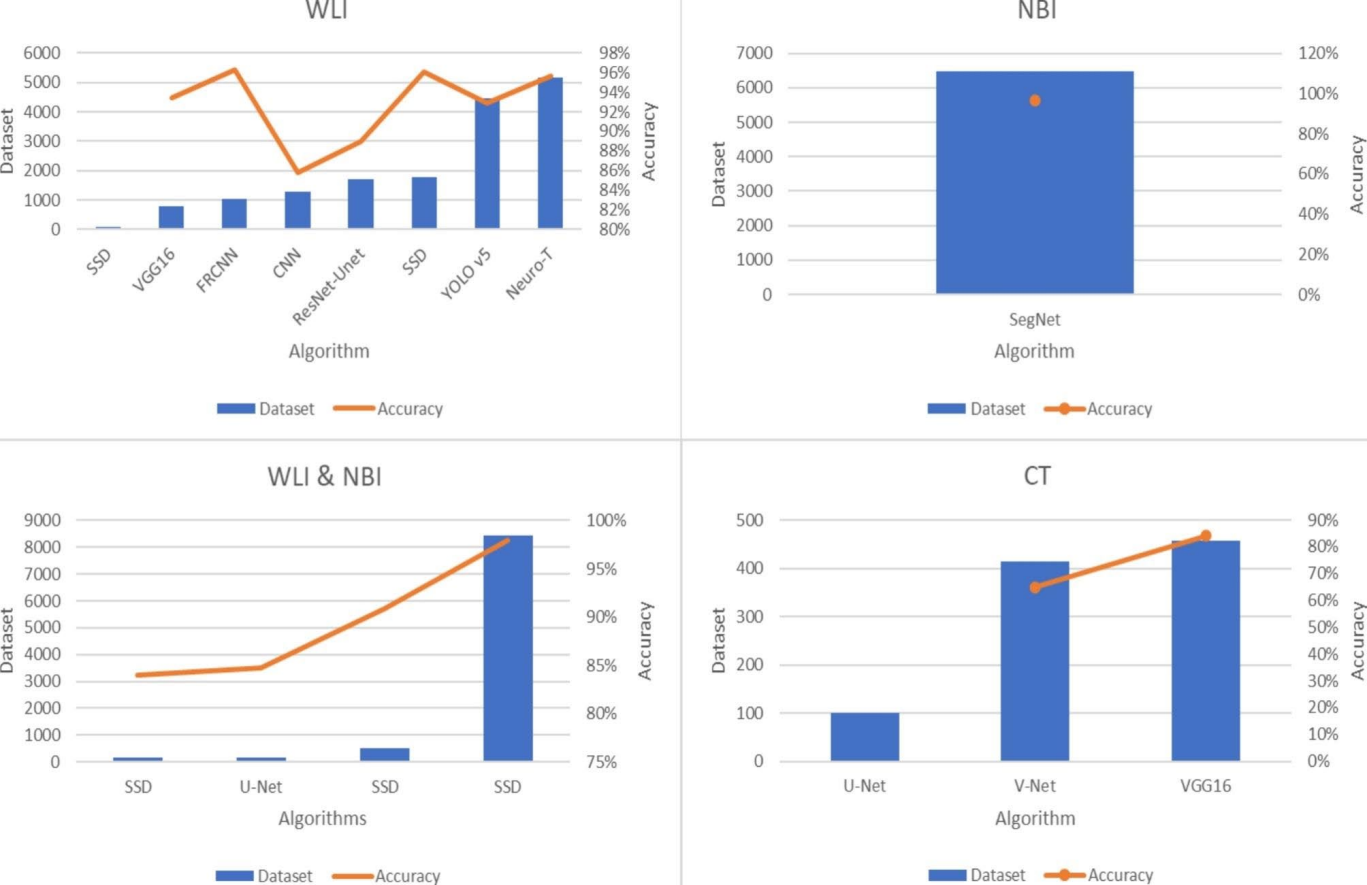
The Dataset type and performance of ML algorithms

Our review identified variations in dataset types and sizes used for identifying and diagnosing esophageal cancer, as well as differences in performance levels among various ML algorithms. The lowest dataset size of 100 images was associated with CT and WLI modalities, utilized by the U-Net and SSD algorithms [13, 38], respectively (Table 2), while the largest dataset size was observed in the combination of WLI and NBI modalities, comprising 8,428 images, and utilized by the SSD algorithm [20] (Fig. 8). In processing CT images, the V-Net algorithm achieved 65% accuracy with a dataset size of 414 images, while the VGG16 algorithm achieved 84.2% accuracy with 457 images. No results were reported for the U-Net algorithm with a dataset size of 100 images [38, 40, 41].

**Table 2:** The Dataset type and performance of ML algorithms

Modality	Algorithm	Dataset	Accuracy
CT	U-Net	100	-
CT	V-Net	414	65%
CT	VGG16	457	84.20%
WLI	SSD	100	-
WLI	VGG16	805	93.43%
WLI	FRCNN	1051	96.29%
WLI	CNN	1272	85.83%
WLI	ResNet-Unet	1704	89%
WLI	YOLO v5	4447	92.90%
WLI	Neuro-T	5162	95.60%
WLI	SSD	1780	96.10%
NBI	SegNet	6473	96.50%
WLI & NBI	SSD	8428	98%
WLI & NBI	SSD	155	84%
WLI & NBI	SSD	498	90.90%
WLI & NBI	U-Net	165	84.72%

The SSD algorithm was employed in two studies for processing the WLI dataset [13, 30] and the combination of WLI & NBI in three studies [20, 25, 37]. Our study revealed a relationship between accuracy and sample size for the SSD algorithm in image processing. Specifically, in WLI & NBI images, the value of accuracy and sample size are equal to respectively 84%, 90.9%, 98% and 155, 498, 8428 and for WLI images are equal to 96.1% and 1780 .In another study, accuracy ranged from 96.1-1,780%[20, 25, 37]. This relationship was also observed in other algorithms with different modalities [27, 29, 33]. However, due to a lack of data, it was not feasible to compare the performance of the algorithm for a specific modality with varying sample sizes. The dataset size can be compared with corresponding accuracy, as outlined in Table 2.

**Fig. 9:**
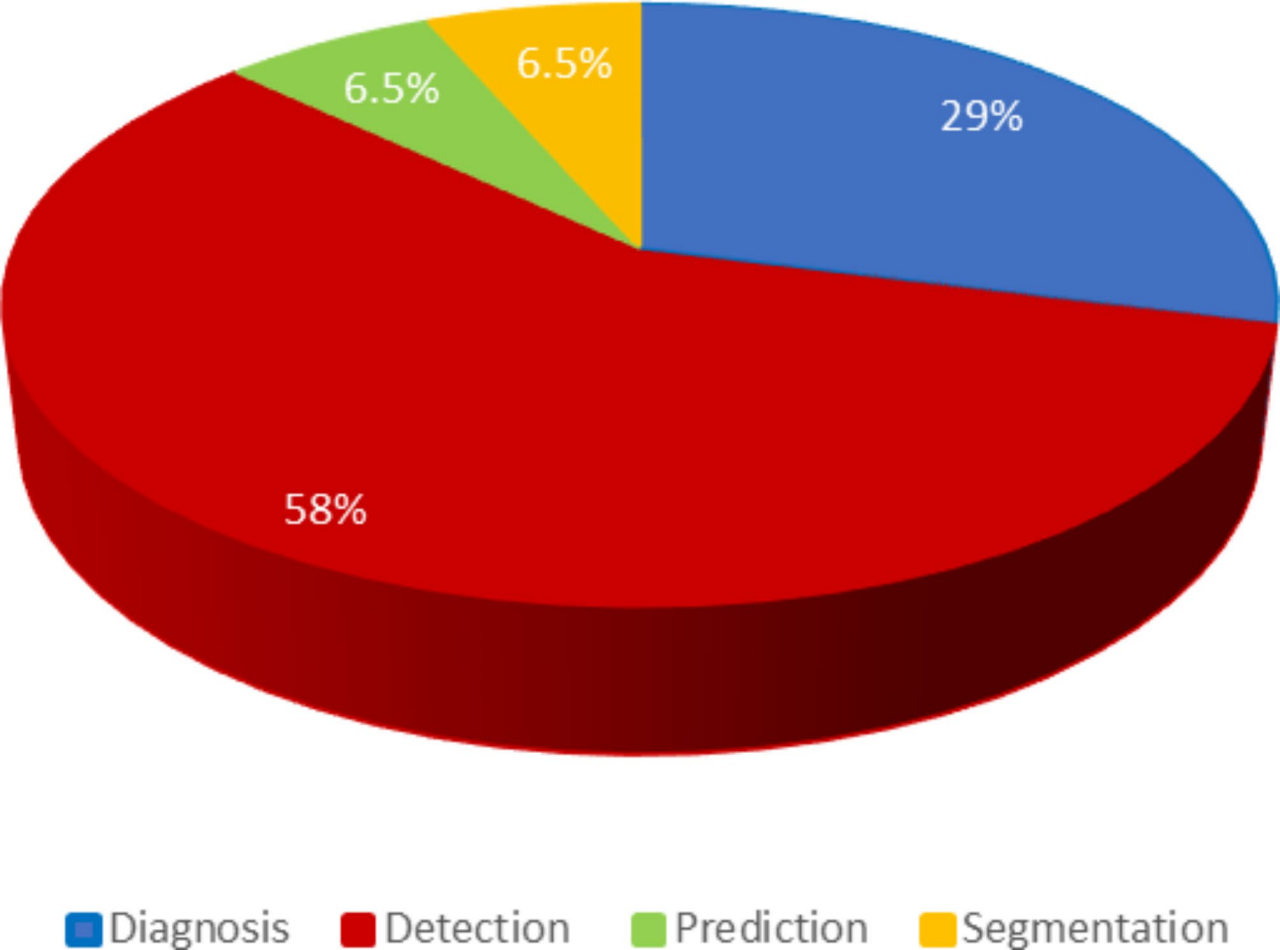
Classification of studies based on their methodologies

**Fig. 10:**
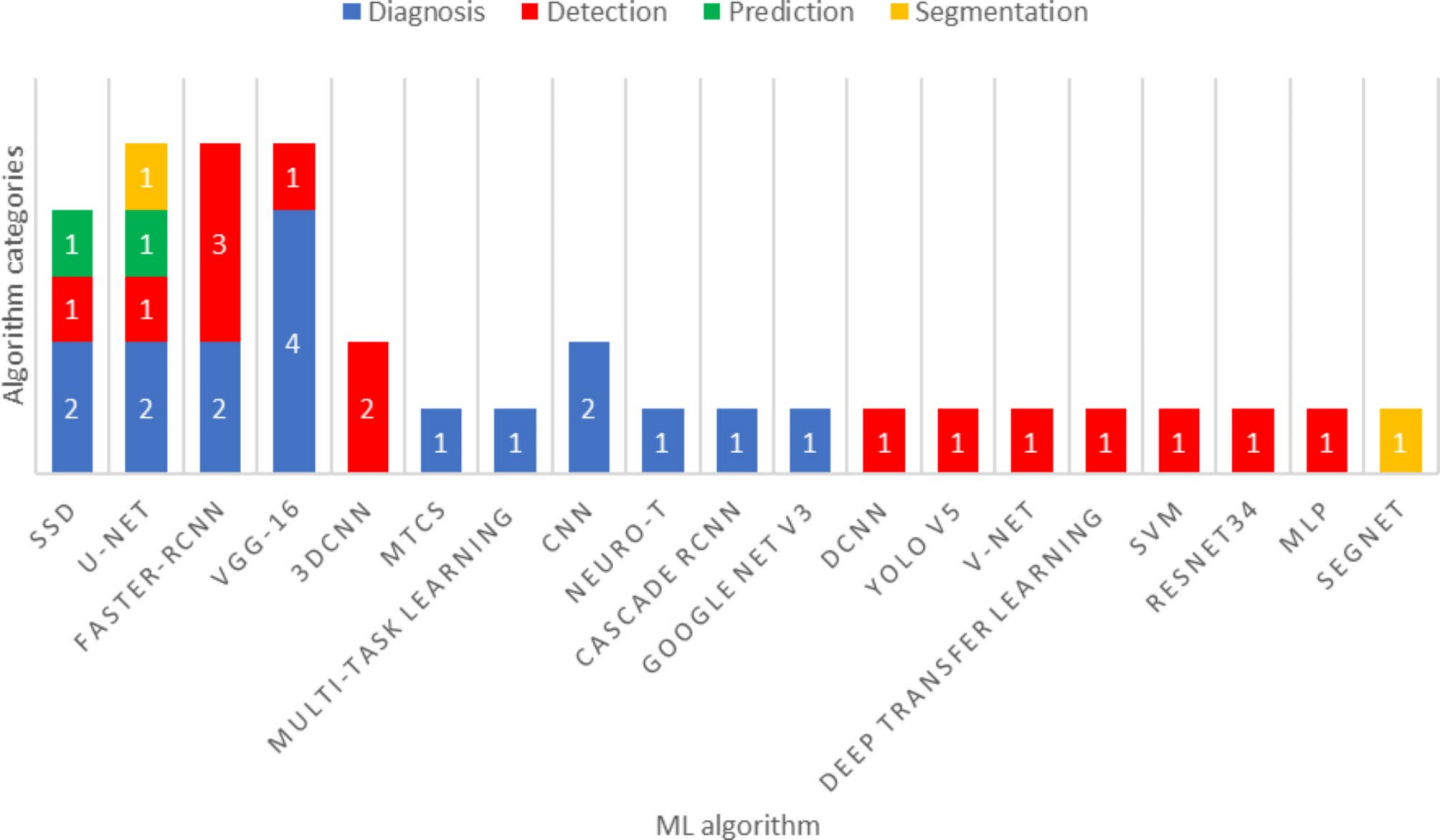
Classification of type ML algorithms used for Diagnosis, Detection, Prediction, and Segmentation

Several studies have employed AI using ML or DL algorithms to assess their accuracy in diagnosing or classifying EC. A majority of these algorithms have utilized endoscopic images for detecting, diagnosing, and classifying cancer and esophageal neoplasms through automatic feature selection and self-learning techniques [23]. Based on the methodology of the reviewed studies, they can be categorized into the following four groups, as presented in Figs. 9 and 10 also presents classification of the ML algorithms used for diagnosis, detection, prediction, and segmentation .

1. Diagnosis (identification and classification) (n = 9) [26, 28, 31, 34, 35, 41–43, 48].
2. Detection (n = 18) [13, 19, 20, 25, 27, 29, 32, 33, 37, 40, 44–47, 49–52].
3. Prediction (n = 2) [30, 36].
4. Segmentation (n = 2) [38, 42].

#### Diagnosis

In this section, all studies with the exception of Putten et al. [35], Guo et al. [42] and Ghatwary et al. [48] investigated both types of EC, including EAC and ESCC. Moreover, all studies employed a two-step process for cancer diagnosis, comprising of segmentation or identifying the areas associated with abnormality and classification. Of the 9 studies reviewed, 6 employed various CNN algorithms for both segmentation and classification. The remaining 3 studies utilized other ML algorithms, including one studies of the MTL algorithm [34], one study of the transfer learning [31], and one study that used Google Net and TensorFlow algorithms [43]. Among the studies, the highest accuracy was achieved by Alharbe et al. [31] with a value of 99.7%, utilizing the ResNet101 and Feed Forward Neural Networks (FFNN) algorithms for segmentation and classification. Conversely, the lowest accuracy was reported by Sui et al. [41] with a value of 65%, using the V-Net algorithm.

#### Detection

This section reviews 18 studies focused on the application of ML algorithms to detect EC. Unlike the diagnosis section, where separate steps were employed for processing and obtaining results, the algorithms used in this section utilize object detection methods, presenting the results to the user in a single processing step. Of these studies, two focused specifically on the detection of EAC, with Sali et al. utilizing the 34ResNet ML algorithm with 387 images [46] and Groof et al. employing the hybrid ResNet-Unet algorithm with 1704 images [33]. Three studies specifically investigated the detection of ESCC using SSD, YOLO v5, and DCNN algorithms [20, 29, 47], while the remaining studies utilized various CNN-type algorithms to investigate the detection of both EAC and ESCC. Only Gong et al. utilized the Neuro-T algorithm, achieving the highest accuracy level of 95.6% with the No-code deep-learning tool “Neuro-T” algorithm [27]. Wang et al., on the other hand, reported the lowest accuracy rate of 83% using the Cascade RCNN algorithm [13, 19, 25, 32, 37, 40, 44, 45, 49–52].

#### Prediction

Two studies utilized ML methods to predict EC in its early stages and take timely action for treatment. Tsai et al. applied the SSD algorithm to 1780 images and reported 96.1% accuracy in the early detection of EC. The authors emphasized that the SSD method can predict both types of EC in the early stages [25]. In another study, Fang et al. achieved an 84.72% accuracy rate using the U-Net algorithm with NBI images, and an 82.38% accuracy rate using WLI images. The authors demonstrated the potential of ML techniques in improving the accuracy of EC detection, particularly in the early stages [36].

#### Segmentation

Guo et al. used the SegNet algorithm with 6473 images for the automatic real-time segmentation of precancerous lesions and ESCC to aid in EC diagnosis [42]. In another study, Lou et al. reported that using the U-Net algorithm, a subtype of NN, they were able to perform segmentation in both types of EC (EAC & ESCC). These studies showed that the results obtained in both studies are acceptable [38].

Regarding the processing time of DL algorithms, we reviewed studies that provided information on the required time or processing speed of the utilized algorithms. Among these studies, the most frequent information was available for the Faster R-CNN, U-Net, and SSD algorithms. Specifically, considering the processing of white light imaging (WLI) images, it was observed that the SSD algorithm processed the image and provided the result within a range of 0.1–0.2 s, while U-Net required approximately 46.3 s. For the R-CNN algorithm, the processing time ranged from 13.38 to 37.81 s, whereas the Fast R-CNN algorithm operated within a range of 0.65–2.1 s. Lastly, the Faster R-CNN algorithm exhibited a processing time of 0.3–0.45 s [13, 33]. Further details regarding the processing time of the reviewed algorithms can be found in Table 3.

**Table 3:** Processing time of DL algorithms

Algorithm	Time	Modality	Type
Faster R-CNN	74 s	-	segmentation
Faster R-CNN	5.3 s	-	Detection
U-Net	4.24 s	CT	segmentation
CNN	113 frames/s	ultrasound endoscopy	Prediction
Cascade RCNN	42 frames/s	ultrasound endoscopy	Prediction
U-Net	46.3 s	WLI	Detection
R-CNN	13.38–37.81 s	WLI	Detection
Fast R-CNN	0.65–2.1 s	WLI	Detection
Faster R-CNN	0.3–0.45 s	WLI	Detection
SSD	0.1–0.2 s	WLI	Detection
SSD	1.0 s	WLI&NBI	Detection

Table 4 provides a summary of the performance data of the reviewed studies for early detecting of EC using ML techniques. The studies highlighted the potential of DL approaches in improving segmentation accuracy, which is a critical step towards enhancing the accuracy of EC detection and ultimately improving patient outcomes.

**Table 4:** Performance data on studies for early detection of EC using a ML

Author	Cancer Type	Modality	algorithm	image	patient	AUROC	accuracy	sensitivity	specificity
Lou et al.	EAC & ESCC	CT	U-Net	80	-	-	-	-	-
Ghatwary et al.	EAC & ESCC	WLI	Faster R-CNN SSD	100	39	-	-	96%	92%
Tang et al.	EAC & ESCC	WLI	MTCS	805	255	-	93.43%	92.82%	96.20%
Ghatwary et al.	EAC	-	Faster R-CNN	1000	-	-	-	-	-
Yu et al.	EAC & ESCC	endoscopy images	MTL	1003	-	-	96.96%	95.64%	97.70%
Wu et al.	EAC & ESCC	WLI	Faster-RCNN DSN	1051	-	-	96.28%	90,34%	97,18%
Liu et al.	EAC & ESCC	WLI	CNN	1272	748	-	85.83%	94.23%	94.67%
Groof et al.	EAC	WLI	hybrid ResNet-Unet	1704	669	-	89%	90%	88%
Tang et al.	ESCC	-	DCNN	4002	1078	95,4%	91.30%	97.9%	88.6%
Meng et al.	ESCC	WLI	YOLO v5	4447	837	98,2%	92.9%	91.90%	94.7%
Gong et al.	EAC & ESCC	WLI	No-code deep-learning tool “Neuro-T” version 2.3.2	5162	-	95%	95.6%	-	-
Shiroma et al.	ESCC	WLI & NBI	SSD	8428	-	-	98%	100%	100%
Du et al.	EAC & ESCC	-	RWS ECA-DDCNN	20,965	4,077	98.77%	90.63%	-	-
Putten et al.	EAC	endoscopy images	U-Net Transfer Learning	494,356	-	-	87.50%	92.50%	82.50%
Gan et al.	EAC & ESCC	OCT image	D-UCN	-	-	-	98%	-	-
Wang et al.	EAC & ESCC	endoscopy & ultrasound	Cascade RCNN	-	80	-	83%	-	-
Sui et al.	EAC & ESCC	CT	V-Net	-	414	-	65%	88.80%	90.90%
Takeuchi et al.	EAC & ESCC	CT	CNN- VGG16	-	457	-	84.20%	71.70%	90.00%
Ghatwary et al.	EAC & ESCC	video	3DCNN	-	-	-	91.10%	-	-
Alharbe et al.	EAC & ESCC	image	Deep transfer learning	-	-	-	99.7%	99.49%	99.78%
Zhao et al.	EAC & ESCC	digestive endoscopy	Google Net V3 TensorFlow 1.6	-	300	91%	91.00%	90.00%	92.0%
Collins et al.	EAC & ESCC	-	SVM, MLP, 3DCNN	-	10	93%	-	-	-
Zhao et al.	EAC & ESCC	-	CNN	-	500	-	-	98%	99,6%
Chen et al.	EAC & ESCC	-	Faster RCNN	1520	421	-	92.15%	-	-
Tsai et al.	EAC & ESCC	WLI & NBI	SSD VGG-16	155 153	-	-	86%	92%	-
Tsai et al.	EAC & ESCC	WLI	SSD	1780	-	-	96.1%	81.6%	-
Sali et al.	EAC	whole-slide tissue histopathology images (WSIs)	ResNet34	387	130	-	-	-	-
Wang et al.	EAC & ESCC	WLI & NBI	SSD	498 438	-	-	90.90%	96.20%	70.40%
Zhang et al.	EAC & ESCC	-	Faster R-CNN VGG16	6445	200	-	90.3%	92.5%	88.70%
Guo et al.	ESCC	NBI	SegNet	6473	-	-	-	98.04%	95.03%
Fang et al.	EAC & ESCC	WLI & NBI	U-Net	75 91	-	-	84.72%	-	-

## Discussion

EC is a highly lethal malignancy, with a 5-year survival rate of less than 20%, mostly due to late diagnosis and treatment [53, 54]. Endoscopic ultrasound, a commonly used diagnostic method, has limited sensitivity in detecting small-sized lesions, which can impact diagnostic accuracy [33]. In recent years, researchers have explored novel non-invasive imaging methods such as radiomics, aimed at improving the diagnosis and treatment of EC. Additionally, the use of ML technology in the analysis and interpretation of clinical images has shown potential in providing valuable information for the early detection of EC. Therefore, this study aimed to conduct a systematic review of the literature to investigate the use of ML in the early detection of EC. By synthesizing the findings from previous studies in the field, this study aimed to address critical relevant research questions regarding ML methods and provide insights into their potential in improving the accuracy and effectiveness of EC early detection.

Our systematic review highlights the significance of imaging techniques in achieving more accurate detection of EC at an early stage. For instance, the accuracy of CT imaging was found to be lower than that of other modalities, at 82.37%. Additionally, while the NBI method was only accurate in detecting ESCC, the WLI method, with a diagnostic accuracy of 96.1%, was found to be more effective in detecting both EAC and ESCC [30, 40, 42]. Despite the possibility of faults in the detection and estimation of cancer grading through WLI images due to the delicate and imperceptible mucosa and surface lesions of the esophagus, WLI images were among the top three modalities in terms of the accuracy of results in early detection of EC using ML [55]. Our findings suggest that the choice of imaging technique is a crucial factor in improving the accuracy of early EC detection, and further studies could benefit from optimizing the use of these techniques in combination with ML algorithms.

Accuracy of EC detection using ML methods is highly dependent on the type of algorithms used and the quality of data used for training. In particular, DL methods, especially CNN-based algorithms, outperform other ML models such as SVM and MLP in terms of detection accuracy, sensitivity, specificity, and AUROC indicators. Furthermore, it appears that the use of combined methods and multiple steps in machine and DL algorithms produces better results than other approaches [33]. For example, Alharbe et al.(2022) developed a deep transfer learning-driven hybrid algorithm for the classification of EC, which utilized multiple algorithms, including ResNet, DCNN, and Gaussian filtering, for data preprocessing, feature extraction, and EC detection. This approach achieved an accuracy of 99.7%, a sensitivity of 99.49%, and a specificity of 99.78%, which demonstrated a significant improvement in detection accuracy compared to other algorithms [31]. Similarly, the combination of U-Net and transfer learning methods for the early detection of EC resulted in superior outcomes, with 87.50% accuracy, 92.50% sensitivity, and 82.50% specificity [35]. In general, adopting a set of combined approaches in preprocessing and detection tasks based on EC images may help to reduce errors in the diagnosis of EC, which can potentially assist clinicians in the early diagnosis of EC, thereby reducing the mortality rate among patients with EC. This finding underscores the importance of data quality, algorithm selection, and preprocessing methods in developing effective ML-based detection models for EC [37, 44].

To ensure the generalizability of the results, large training datasets are often essential for the training, validation, and testing of ML algorithms, particularly in clinical settings. ML applications are known to benefit from large sample sizes as they help minimize bias. However, smaller sample sizes can sometimes result in higher accuracy, which has been observed in the reviewed studies [28, 34]. Interestingly, studies that reported the highest accuracy did not provide information on the sample size used, which suggests that other factors such as feature processing and model parameter tuning may also play a crucial role [31, 39, 53]. Therefore, future studies should aim to investigate the optimal sample size for issues related to the clinical field, while also examining the characteristics of ML algorithms, including feature extraction, selection, and optimization, to achieve more accurate and reliable results. By taking a comprehensive approach, we can advance our understanding of ML applications and improve their efficacy in medical imaging and diagnosis.

Furthermore, choosing the type of dataset and modality can affect the performance of ML algorithms in medical imaging. Our study revealed that the SSD algorithm achieved higher accuracy in processing WLI & NBI images than WLI images [20, 30], while similar results were observed for VGG16 algorithm in processing CT and WLI images [28, 40]. However, these results should be interpreted while considering the dataset volume. Although a direct relationship between sample size and accuracy was observed, our study showed that the dataset type is another important factor in achieving high performance. Therefore, future studies should examine ML algorithms under the same conditions of sample size and dataset type to obtain more reliable results. This limitation of our study emphasizes the importance of using consistent conditions in dataset selection to evaluate the performance of ML algorithms accurately and reliably, thereby advancing our understanding of their applications in medical imaging and diagnosis.

ML relies on several components such as dataset, algorithms and models, feature extraction, and training, all of which contribute to the performance of the models [54, 55]. Our systematic review revealed that the U-Net, Faster R-CNN, and SSD algorithms are the most frequently used among the studies conducted for the early detection of EC The results indicated that the performance of U-Net and Faster R-CNN algorithms was comparable in terms of accuracy, regardless of the number of samples used [13, 26, 35, 36]. However, significant differences were observed with the SSD algorithm, indicating its sensitivity to the number of samples [20, 25]. Hence, the number of samples used could introduce bias in the study, and further investigations are required to address this issue in future studies.

The results of included studies showed a significant improvement in the performance of segmentation algorithms, specifically U-Net, SegNet, and Transfer learning, in detecting EC with accuracies of 99.7%, 97%, 96%, and 98% obtained [26, 31, 34, 39]. U-Net was found be effective to work with limited training samples in segmentation tasks. It also preserves the complete context of input images by performing classification on each pixel, generating segmentation maps directly in an end-to-end pipeline. This approach is critical in maintaining complete context compared to patch-based segmentation approaches [59]. However, U-Net’s large number of parameters due to skip connections and additional layers in the expanding path may make the model more prone to overfitting, especially when working with small datasets. On the other hand, SegNet uses less memory by transferring only the pooling indices from the compression path to the expansion path, but may lose neighboring information when unpooling from low-resolution feature maps [42].

Our study also demonstrated that the use of proposed segmentation algorithms in the structure of transfer learning can increase their performance to an acceptable level for segmenting EC images [35]. Transfer learning is a ML technique that applies knowledge gained from one problem to another similar task or domain, and CNN models can be trained either from scratch or through transfer learning [60]. In future studies, it is suggested to investigate the challenges in improving transfer learning performance in the field of EC by using the combination of effective algorithms in segmentation and classification. Overall, our study highlights the potential benefits of segmentation algorithms and transfer learning in improving the accuracy of EC detection.

Faster R-CNN and SSD were the most commonly used algorithms for object detection according to the results of this review. Faster R-CNN is a DL model known for its superior performance and efficiency in object detection, utilizing a novel region proposal network to generate region proposals quickly and accurately [56]. It extracts fixed-size feature maps from medical images [57], assigns classes, and predicts bounding boxes in a single run, making it an efficient and effective tool for object detection [58]. Faster R-CNN’s advantages include higher detection quality than other CNN-based methods, single-stage training, and no requirement for disk storage for feature caching [59]. The SSD is a DL approach that employs a feed-forward CNN to produce a fixed-size array of bounding boxes and scores. These scores indicate the presence of object class instances within the respective boxes, followed by a non-maximum suppression step to generate the final detections [60]. The SSD approach stands out from other object detection algorithms as it can detect multiple objects present in an image in a single shot using a multibox, thus significantly improving speed without sacrificing accuracy [61, 62]. By utilizing multiple convolutional layers, the SSD algorithm detects objects with higher robustness to scale changes, but it may miss small objects, which is a notable limitation [62].

In the early detection of esophageal cancer, only two studies were found in the segmentation category, despite its crucial role in image processing. This raises questions about the potential of segmentation compared to other categories for enabling early detection of EC. Can accuracy and precision be improved by combining and using more comprehensive methods? Similarly, only two studies utilized prediction, indicating a limitation in collecting longitudinal data, particularly in patients with esophageal cancer. The necessary prognosis does not occur until after the patient is infected, which may explain the limited use of relevant algorithms in this field. To overcome this challenge, further research is needed to explore the potential of combining segmentation and prediction methods and to collect longitudinal data in patients with esophageal cancer. Such efforts will improve the accuracy and effectiveness of early detection and contribute to the development of more advanced ML algorithms.

In terms of processing speed, the SSD algorithm outperformed the U-Net and Faster R-CNN algorithms [63, 64], and in terms of accuracy, the SSD algorithm demonstrated the highest level of accuracy [20]. Thus, the choice of these algorithms in studies could be attributed to their superior performance in terms of accuracy and speed, which warrants further investigation in future studies. Overall, the selection of the appropriate ML components is crucial for the accuracy and efficiency of the models, and researchers should carefully consider these factors when designing studies for the early detection of EC using ML.

Time is an important factor in the early detection of EC during real-time imaging, such as real-time endoscopy surveillance. The application of ML methods in real-time detecting EC can support clinical experts to focus on or examine the suspicious area faster and avoid diagnostic errors. Therefore, processing speed and response can be critical factors in evaluating the performance of ML methods. Previous studies have highlighted the importance of computational speed in real-time endoscopic surveillance. For example, Groof et al.(2020) designed an algorithm for real-time early detection of EC in classification tasks and analysis of endoscopic images, achieving a computational speed of 0.24 s, which although still relatively slow for DL systems, is suitable for use during real-time endoscopy surveillance [33]. Several studies have also implemented specific techniques to optimize the performance of CNN and RCNN methods in real-time to improve detection accuracy and speed. Wang et al.(2021) investigated the performance of CNN and Cascade RCNN algorithms in online cancer diagnosis and showed that the operation speed of the Cascade RCNN model improved. Such approaches have been reported as useful strategies to increase algorithm performance, where the CNN prediction speed was 113 fps and the Cascade RCNN model was 42 fps [19]. Similarly, the application of the Deep CNN (DCNN) algorithm by Tang et al. showed that with the correct adjustment of the algorithm and its parameters, the response rate of DCNN only needs 15 milliseconds to detect esophageal lesions in each image [47]. However, there is limited studies on the characteristics and settings of real-time detection systems for EC requiring future studies to investigate the characteristics, settings, and hardware of online systems for detecting EC.

Advanced ML, particularly DL, is a rapidly evolving technology that is becoming increasingly widespread in various fields. Compared to traditional ML, which relies on experience to improve system performance, data-driven ML utilizes large datasets to identify patterns and predict future outcomes. While large datasets are often considered necessary for successful deep learning applications, techniques such as transfer learning can enable deep learning even with limited data sets [65]. Recent studies have demonstrated the potential of advanced ML techniques in the field of EC diagnosis, including the use of artificial images and generative adversarial network (GAN) and variable autoencoder (VAE) models to improve image quality and DL performance [66]. The use of autoencoders and long short-term memory (LSTM) networks has also shown promise in detecting esophageal abnormalities and improving classification performance [45, 67, 68]. Future research should continue to explore novel applications of advanced ML techniques and focus on combined pre-processing and classification systems to enhance accuracy and effectiveness in EC diagnosis.

## Conclusion

Early detection of EC is crucial for improving the prognosis and survival rate of patients. Unfortunately, traditional diagnostic methods are often not able to detect the disease in its early stages. ML has emerged as a powerful tool for improving the accuracy of medical diagnosis. Our systematic review highlights the potential of ML techniques in the early detection of EC using non-invasive imaging methods such as CT scans and endoscopic images. The performance of DL algorithms, especially CNN based methods, has shown to be superior to other ML models such as Support Vector Machine (SVM) and Multilayer Perceptron (MLP). Moreover, the selection of appropriate algorithms, data sets, feature extraction, and training are crucial components that affect the performance of ML models. The use of combined approaches and multiple steps in ML and DL algorithms have shown better results in detecting EC. Furthermore, the processing speed and response time of ML models can be critical factors in real-time endoscopy surveillance. In conclusion, the application of ML techniques in the early detection of EC holds great promise in improving patient outcomes. Future studies should focus on optimizing the performance of ML models, investigating the characteristics and settings of real-time diagnostic systems for EC, and exploring the use of non-invasive imaging methods for early detection.

## Data Availability

The datasets used and/or analyzed during the current study are available from the corresponding author on reasonable request.

## List of abbreviations

CT: Computed Tomography
CAD: Computer-Aided Diagnosis
CNN: Convolutional Neural Networks
DCNN: Deep CNN
DL: Deep Learning
D-UCN: Dual-Stage U-shape Convolution Network
DSN: Dual-Stream Network
EAC: Esophageal Adenocarcinoma
EC: Esophageal Cancer
ESCC: Esophageal Squamous Cell Carcinoma
ML: Machine Learning
MLP: Multilayer Perceptron
MTCS: Multi-Task Classification and Segmentation
MTL: Multi-Task Learning
NBI: Narrow-Band Imaging
NN: Neural Networks
OCT: Optical Coherence Tomography
RWS: Random Weighted Sampling
R-CNN: Region-Based CNN
SSD: Single-Shot Multibox Detector
SVM: Support Vector Machine
WLI: White Light Imaging
WSTHI: Whole-Slide Tissue Histopathology Images
